# Molecular markers enhance substantially the distinctness of alfalfa varieties for registration and protection

**DOI:** 10.1002/tpg2.20556

**Published:** 2025-02-05

**Authors:** Paolo Annicchiarico, Nicolò Franguelli, Barbara Ferrari, Giacomo Campanella, Stefano Gualanduzzi, Margherita Crosta, Chiara Delogu, Giorgia Spataro, Nelson Nazzicari

**Affiliations:** ^1^ Council for Agricultural Research and Economics (CREA) Research Centre for Animal Production and Aquaculture Lodi Italy; ^2^ CREA Research Centre for Plant Protection and Certification Bologna Italy; ^3^ CREA Research Centre for Plant Protection and Certification Milano Italy

## Abstract

Plant varieties must satisfy distinctness, uniformity, and stability (DUS) requirements for registration. Morphophysiological trait‐based distinctness may be challenging for cultivars of major perennial forages. Our study focused on alfalfa (*Medicago sativa* L. subsp. *sativa*) with the aims of (a) comparing morphophysiological distinctness with molecular distinctness based on genotyping‐by‐sequencing (GBS) or the alfalfa DArTag panel, envisaging different statistical criteria for molecular distinctness, and (b) assessing the consistency of morphophysiological and molecular cultivar diversity. The 18 most grown Italian varieties were jointly reevaluated morphophysiologically and were characterized molecularly using three bulked DNA samples of 200 independent genotypes per cultivar. Morphophysiological distinctness was limited by correlations between traits and resulted in 39 non‐distinct cultivars in 153 paired comparisons and three cultivars distinct from any other. Best configurations for molecular distinctness featured about 10‐fold more polymorphic markers and 10‐fold lower average read depth per marker for GBS compared to DArTag. DArTag markers allowed for somewhat better variety distinction than GBS. They reduced to 11 the non‐distinct cultivars in paired comparisons and increased to 11 the completely distinct cultivars, based on a principal components analysis of allele frequencies followed by analyses of variance on cultivar principal component scores. This criterion achieved greater variety distinctness than cluster analysis with bootstrap values, discriminant analysis, or analysis of molecular variance. Morphophysiologically distinct cultivars were generally distinct molecularly, but not the reverse. Mantel's test revealed a modest consistency across morphophysiological and DArTag (*r* = 0.39) or GBS‐based (*r* = 0.46) measures of cultivar Euclidean distance. Our results and other considerations strongly encourage the adoption of molecular distinctness for alfalfa DUS.

## Core Ideas

- Poor morphophysiological distinctness limits the alfalfa variety registration and protection.
- Molecular markers allow for much greater variety distinctness than morphophysiological traits.
- The alfalfa DArTag panel is preferable to genotyping‐by‐sequencing for variety distinction.
- Morphophysiologically distinct cultivars usually are molecularly distinct (but not the reverse).
- There is modest consistency of morphophysiological and molecular measures of cultivar diversity.

**Abbreviations**
AMOVA: analysis of molecular variance
ANOVA: analysis of variance
DUS: distinctness, uniformity, and stability
GBS: genotyping‐by‐sequencing
MDS: multidimensional scaling
PBR: plant breeders’ rights
PC: principal component
PCA: principal components analysis
UPOV: International Union for the Protection of New Varieties of Plants
VCU: value for cultivation and use

## 1 INTRODUCTION

The recognition of plant breeders’ rights (PBR) is fundamental to reward a time‐ and resource‐consuming activity such as plant breeding and thereby support a sustainable increase in crop productions. An international regulation for PBR was enforced by the convention that was established by UPOV (International Union for the Protection of New Varieties of Plants) in 1968 and amended in 1972, 1978, and 1991, which is currently recognized by 76 countries. The convention sets the criteria for variety registration and protection and the conditions for the use of registered varieties as a genetic resource (UPOV, 1991). Candidate varieties are granted registration only if they satisfy distinctness, uniformity, and stability (DUS) criteria. Variety distinctness, which is the main DUS requirement, is also important for authorities that control and certify seed production chains and in lawsuits regarding illicit seed marketing, variety plagiarism, and essential derivation claims (Van Wijk & Louwaars, 2014). According to UPOV (1991), a variety “shall be deemed to be distinct if it is clearly distinguishable from any other variety whose existence is a matter of common knowledge at the time of the filing of the application.” The distinctness requirement is satisfied if at least one statistically significant difference can be observed between the candidate variety and each of the registered cultivars across a set of species‐specific morphophysiological characteristics that do not need to be of agronomic value (UPOV, 2015). In the EU, where registration in the national list of one member state is mandatory for variety marketing, candidate varieties have to meet not only the DUS requirements but also sufficient value for cultivation and use (VCU).

Variety distinctness based on morphophysiological traits was initially conceived in a period featuring fairly small numbers and long commercial lifespan of the registered varieties. Concern has been expressed in the last decades about the duration and the costs of the DUS assessment, which requires the observation of a number of traits under field conditions over at least 2 years on at least 60 spaced plants per candidate or control variety. This approach entails high registration fees, may delay the marketing of varieties, and is difficult to apply for forensic studies or the timely control of seed production chains during the seed certification process (Gilliland & Gensollen, 2010; Jamali et al., 2019, 2020). As a matter of fact, DUS requirements for a candidate variety are actually verified with respect to a small number of registered varieties, which are four for alfalfa (*Medicago sativa* L. subsp. *sativa*) in Italy, to limit the variety registration costs. Another concern regards the possible inability of the morphophysiological trait‐based distinctness to distinguish the large and ever‐increasing number of varieties selected for each species. This limitation may be critical for species bred as synthetic varieties of major forage crops, such as alfalfa and perennial (*Lolium perenne* L.) or Italian ryegrass (*Lolium multiflorum* Lam.) (Gilliland et al., 2020; Mendler‐Drienyovszki et al., 2024). Variety distinction in perennial forages is complicated by substantial intra‐variety variation, which may exceed the inter‐variety variation for many morphophysiological traits (Annicchiarico et al., 2015; Herrmann et al., 2018). For alfalfa, which is the most grown perennial legume worldwide (Annicchiarico et al., 2015), the EU database of registered varieties reports 428 cultivars up to January 2023 (EUPVP, 2024), of which 146 were bred in Italy. Around 1000 alfalfa cultivars are listed in the OECD catalogue (OECD, 2024). Lack of distinctness has already led to the rejection of many alfalfa candidate varieties that exhibited sufficient VCU for registration, thereby nullifying breeders’ selection investments and the agronomic progress that they may have brought. For example, 12 out of 46 candidate varieties proposed in France over the period 2001–2010 were rejected essentially because of insufficient distinctness while featuring sufficient VCU (V. Gensollen, M.‐C. Gras and B. Julier, personal communication, 2016). Likewise, over 12% of the candidate varieties of ryegrass and white clover (*Trifolium repens* L.) were rejected for insufficient distinctness in the UK over the period 2000–2008, reaching 19% in 2008 (Gilliland & Gensollen, 2010). The number of rejected cultivars may be much higher if the distinctness of each candidate variety was actually tested with respect to the whole set of registered varieties. Achieving sufficient distinctness may be especially challenging for varieties issued by recurrent selection, a popular selection scheme whose application has also been proposed for genomic selection (Li & Brummer, 2012).

The increasing difficulty of obtaining sufficient distinctness for agronomically‐valuable candidate varieties and the high cost and time duration of the DUS testing emphasize the potential interest of exploiting molecular marker diversity to assess cultivar distinctness. An additional advantage of marker data is their insensitivity to genotype × environment interactions (unlike quantitative morphophysiological traits). Accordingly, several scientists have proposed the adoption of molecular marker‐based variety distinction in general (Jamali et al., 2019; Poets et al., 2020; Wang et al., 2019; J.‐K. Yu & Chung, 2021) and specifically for forage crops (Annicchiarico et al., 2016; Byrne et al., 2013; Julier et al., 2018; Roldán‐Ruiz et al., 2001; Q. Yu et al., 2022). The proposed molecular fingerprinting is generally based on nuclear DNA, since the DNA from chloroplasts and mitochondria may feature low degree of sequence variation (Nybom et al., 2014; Wang et al., 2019). Molecular distinctness is currently accepted by UPOV only for markers with a proven genetic association with a relevant morphophysiological trait (of which they represent a proxy) and under the condition of using for the molecular assessment the same number of individual plants that is used for the ordinary DUS assessment based on morphophysiological traits (i.e., at least 60 plants) (UPOV, 2020). This scenario is hardly applicable to alfalfa (or other forage crops) because of the limited information on markers associated with distinctive traits and the high cost of molecular analyses performed on many individual plants. Molecular markers are accepted by UPOV also for managing reference collections of registered cultivars aimed to select control varieties for the DUS assessment based on the highest marker‐based similarity with the candidate variety (UPOV, 2020). Such a use requires that the molecular similarity reflects well the morphophysiological similarity of the varieties, an assumption pending verification for alfalfa and other perennial forage species.

**Core Ideas**

- Poor morphophysiological distinctness limits the alfalfa variety registration and protection.
- Molecular markers allow for much greater variety distinctness than morphophysiological traits.
- The alfalfa DArTag panel is preferable to genotyping‐by‐sequencing for variety distinction.
- Morphophysiologically distinct cultivars usually are molecularly distinct (but not the reverse).
- There is modest consistency of morphophysiological and molecular measures of cultivar diversity.

The exploitation of molecular distinctness in the absence of morphophysiological distinctness is a cornerstone of a revision of variety registration procedures for forage crops proposed by Gilliland et al. (2020). The proposed distinctness assessment would focus on the overall molecular diversity across a large number of single nucleotide polymorphism (SNP) markers, rather than on differences for a few markers as envisaged in early studies (e.g., Pupilli et al., 1996). This option would minimize the risk of registration of essentially‐derived varieties selected by a drastic change of allele frequencies for a few markers, which is a major concern associated to the use of molecular distinctness (Gilliland & Gensollen, 2010; UPOV, 2004). For alfalfa, the cultivar distinctness studies by Annicchiarico et al. (2016) and Julier et al. (2018) could rely on thousands of genotyping‐by‐sequencing (GBS)‐generated polymorphic markers. The recently developed open‐access alfalfa DArTag panel of 3000 loci (Zhao et al., 2023) is another option of interest for alfalfa cultivar fingerprinting because of its lower genotyping fees and somewhat easier data management (e.g., in terms of missing data), albeit at the cost of lower marker number in comparison with GBS. Anyway, about 500 SNP markers could be sufficient for alfalfa variety distinction according to results in Julier et al. (2018).

Gilliland et al. (2020) proposed to assess the molecular distinctness of forage crop varieties using genotyping data relative to some independent bulks of plants per cultivar (acting as experiment replicates) rather than many individual plants per cultivar. While both sampling strategies provide consistent genetic information (Julier et al., 2018), the former allows access to a large plant population sample by a modest evaluation cost and can reduce the bias of the sampling effect that arises from rare marker alleles (Pupilli et al., 1996). Variety distinctness may be granted according to a minimal threshold of genetic distance, but this approach, mainly studied for inbred crops (Van Eeuwijk & Law, 2004), would require the prior determination of a reliable, crop‐specific threshold through extensive research work. An alternative avenue for granting molecular distinctness that is perfectly consistent with morphophysiological trait‐based distinctness is the expression of results according to a Type 1 error rate through a statistical test whose conclusions depend on the variation in allele frequencies of cultivar replicates represented by independent bulked plants. In alfalfa and ryegrass, a principal components analysis (PCA) performed on bulked DNA samples of sets of cultivars revealed overall SNP marker variation among cultivar replicates not only when bulking 50–100 plants (Annicchiarico et al., 2016; Julier et al., 2018; Q. Yu et al., 2022) but even when bulking 200 plants (Byrne et al., 2013). In fact, the intra‐variety variation may be substantial also for molecular marker data in forage crops (George et al., 2006; Pupilli et al., 2000). Annicchiarico et al. (2016) envisaged two methods for assessing the molecular distinctness of forage crop varieties, both involving an initial PCA of the bulked DNA samples of the cultivars aimed at dimensionality reduction of the overall variation in marker allele frequencies. One method was based on an analysis of variance (ANOVA) of the cultivar principal component (PC) scores for each PC axis (considered as a distinct variable), while the other envisaged a discriminant analysis of the cultivars based on their PC score values. Julier et al. (2018) envisaged the paired comparisons of alfalfa varieties by an analysis of molecular variance (AMOVA). Q. Yu et al. (2022) considered a set of ryegrass varieties to be molecularly distinct if a hierarchical cluster analysis with bootstrap values was able to separate the replicates of each cultivar into a distinct group.

In the present study, the 18 most grown registered cultivars of alfalfa in Italy were jointly reevaluated morphophysiologically according to ordinary DUS procedures and were characterized molecularly by two genotyping methods, namely, GBS and the alfalfa DArTag panel. The objectives of this study were: (a) to verify the actual morphophysiological distinctness of an important set of commercial varieties when evaluating them jointly; (b) to optimize the ability to distinguish varieties of both genotyping methods; (c) to compare four statistical criteria for the assessment of molecular distinctness; (d) to compare morphophysiological traits, GBS markers and DArTag markers for ability to distinguish varieties, both separately and when combining morphophysiological data with molecular ones provided by either genotyping method; and (e) to assess the consistency of information on cultivar diversity provided by morphophysiological traits, GBS markers and DArTag markers, in the prospect of a marker‐based management of reference collections of varieties.

## 2 MATERIALS AND METHODS

### 2.1 Plant material

Our study was based on morphophysiological and molecular data of the 18 commercial varieties listed in the Italian Register of Varieties that showed the highest quantity of certified seed in Italy in 2015. Seconding the agreements set by the funding projects POVASE and INVITE with the breeders of the varieties, the name of the varieties cannot be disclosed in any scientific publication (to prevent revealing material that could not be distinguished). Therefore, the varieties were currently coded as alphabetic letters for display of results.

### 2.2 Morphophysiological data

The morphophysiological characterization of the 18 varieties was performed by the same scientific and technical staff and in the same location and conditions that are ordinarily adopted for DUS testing aimed to registration of new alfalfa varieties in Italy. The only difference was represented by the adoption of one evaluation trial, instead of two trials sown in subsequent years. The seed of each variety was provided by the breeder and/or the maintainer of the variety. The experiment was established in Bologna (44°29′ N, 11°00′ E, 54 m elevation) in 2017 according to a randomized complete block design with four replications, using plots that included 25 spaced plants per variety. The morphophysiological characterization was consistent with UPOV's recommendations regarding mandatory traits and observation procedures for alfalfa variety registration trials (UPOV, 2005). The following eight traits were recorded on each plant over the 2‐year trial duration: (a) plant growth habit, assessed 2 weeks before the autumn equinox of the first year on a scale ranging from 1 = erect to 9 = prostrate; (b) plant height in spring of the second year (1 month after the beginning of growing); (c) onset of flowering, observed prior to the first harvest of the second year (as number of days from January 1); (d) proportion of plants with very dark blue‐violet flower, and (e) proportion of plants with variegated flower, both observed on a plot basis in the spring of the second year; (f) length of the tallest stem at full flowering stage, recorded in the spring of the second year; (g) plant height 3 weeks after harvesting, observed after the first, second, third and fourth harvest of the second year (averaging the repeated observations prior to statistical analyses); (h) plant height in autumn, 2 weeks after the autumn equinox of the second year. Plant growth habit and plant height 3 weeks after harvesting are not mandatory according to UPOV (2005) but are envisaged by Italian regulations for alfalfa DUS trials. There was an additional trait recommended as mandatory by UPOV, namely, the frequency of plants with cream, white, or yellow flowers, which was neglected because of a lack of variation in the cultivars evaluated in this study.

### 2.3 Molecular marker data

Plant sampling for DNA analyses was performed on young plants grown in a greenhouse. Each cultivar was genotyped using three bulks of 200 independent plants each, obtained by pooling the central leaflet from the first trifoliate leaf of each plant. Genomic DNA was extracted from each of the 54 bulked DNA samples using the DNeasy Plant Mini Kit (Qiagen). Nucleic acid was quantified by a Quant‐iT PicoGreen dsDNA Assay Kit (P7589, Life Technologies) checking its quality by 1% agarose gel electrophoresis. A trial digestion process was carried out on 10% of the DNA samples using the Optizyme EcoRI restriction enzyme (25,000 U, Fisher BioReagents), to compare bands of cut and uncut DNA. The reaction was performed at 37°C for 1 h, and the enzyme was deactivated at 65°C for 20 min.

The GBS‐based characterization of the cultivars was carried out by the Elshire Group Ltd. laboratory (Palmerston North, New Zealand) according to the method described by Elshire et al. (2011), restricting the genomic DNA by the *ApeK*I enzyme (NEB New England Biolabs, R0643L). Modifications to the method concerned the use of 100 ng of genomic DNA and 3.6 ng of total adapters and the library amplification by Kapa Taq polymerase *α* (KAPA Library Amplification Readymix; Kapa Biosystems, KK2611) through 18 PCR cycles. The library was sequenced using an Illumina X Ten platform with 150 bp paired end reads. After demultiplexing, the average number of reads per DNA sample amounted to 3.2 M. The SNP data were aligned along the reference genome published by Long et al. (2022). The SNP calling was then performed using the Legpipe2 pipeline described by Nazzicari et al. (2024).

The DArTag marker‐based characterization of the 54 bulked DNA samples was performed by the Diversity Array Technology Pty Ltd of the University of Canberra, Bruce (Australia), according to the Alfalfa_DArTag_BI_Cornell_University (1.0) tool developed in collaboration by DArT and Breeding Insight at Cornell University (Zhao et al., 2023). Sequencing was performed on Illumina Hiseq2500/Novaseq6000. We received allele counts of the SNP markers aligned along the reference genome after the outsourced SNP calling performed through the DArT P/L's proprietary pipeline (Diversity Arrays Technology, 2024).

Molecular marker data (either from GBS or DArTag) were represented as SNP allele frequencies for each relevant marker. The markers were filtered for minor allele frequency greater than 5% (computed as average frequency over all samples), as well as for different filtering scenarios relative to the minimum number of reads per locus (10, 20, 30, or 40) and maximum allowed missing rate (1%, 5%, 10%, 20%, and 30%), which resulted, overall, in 20 different filtering configurations of genotyping data for each genotyping method (Table S1).

### 2.4 Statistical analysis

All morphophysiological traits observed on individual plants of each plot (growth habit, onset of flowering, plant height, stem length) were previously averaged to obtain an average plot value used for statistical analyses. The occurrence of statistical differences for each morphophysiological trait was verified for each of the 153 possible pairwise comparisons between the 18 cultivars. For plant growth habit, the cultivars were considered to differ when their difference exceeded 1.5 units on the rating scale, according to UPOV's indications for the analysis of quantitative traits collected on a discrete scale (UPOV, 1990). Each of the other traits underwent an ANOVA in which, according to UPOV's (2022) recommendations, a pair of cultivars differed when their difference exceeded Fisher's least significant difference at *p* < 0.01. The ANOVA was performed on data submitted to the angular transformation for the frequencies of very dark blue‐violet flower and variegated flower. Onset of flowering was analyzed as quantitative since the distribution of its residuals from ANOVA tended to be normal according to the Shapiro–Wilk test. After performing the 153 cultivar pairwise comparisons for each trait, we computed the proportion of comparisons that exhibited no statistical difference for any trait (i.e., the undistinguished pairs of cultivars) and those that showed one or more trait differences. Likewise, we verified the number of completely distinct cultivars, that is, the cultivars that differed from any other cultivar for at least one trait in the pairwise cultivar comparisons. Phenotypic correlations between traits of the 18 cultivars were also assessed.

The occurrence of molecular marker‐based statistical differences was assessed separately for each genotyping method (GBS or DArTag), for each of the 153 possible paired comparisons between cultivars. One criterion proposed in Annicchiarico et al. (2016) involved the performance of a PCA on marker data of the 54 bulked DNA samples, followed by a separate one‐way ANOVA for each PC axis performed on cultivar PC scores whose experiment error was represented by the average variation among bulked DNA samples of each cultivar. In accordance with results for morphophysiological traits, two cultivars were considered distinct if they differed at *p* < 0.01 in at least one ANOVA performed on cultivar PC score values. The first ANOVA was performed on data of PC 1, marking as distinct all the pairs that displayed significantly different PC scores. Following ANOVAs progressively assessed the occurrence of significant differences for the other pairs of cultivars for higher‐ranking PC axes until failing to observe cultivar differences for three subsequent PC axes. The normal distribution of cultivar PC scores was verified by the Shapiro–Wilk test. The set of ANOVAs, which defined each of the 153 possible paired cultivar comparisons as distinct or non‐distinct, was repeated for each of the 20 combinations of filtering levels available for each genotyping method. For each genotyping method, the optimal filtering configuration was defined as the one providing the highest number of distinct cultivars in the paired cultivar comparisons. The ability to distinguish cultivars of each method was assessed not only on the basis of the paired cultivar comparisons but also according to the number of completely distinct cultivars (i.e., those that exhibited distinctness in any pairwise cultivar comparison).

A second criterion for assessing the occurrence of molecular marker‐based cultivar differences relied on a linear discriminant analysis as devised in Annicchiarico et al. (2016). For each of the 153 paired cultivar comparisons, we performed a separate PCA aimed at dimensionality reduction on the bulked DNA samples, retaining the first five PC axes (given the six observations per comparison). Then, we performed a discriminant analysis using two samples per cultivar for constructing the discriminant function and one for validation. The analysis was repeated for all possible combinations of bulked DNA samples (so that each sample was predicted once). Two cultivars were considered distinct if the model was able to correctly classify all six samples. The analysis was performed considering an increasing number of PC axes from one to five and was repeated for all the 20 filtering configurations, retaining the most discriminating configuration for each genotyping method. Also here, we computed the number of distinct cultivars in paired cultivar comparisons and that of completely distinct cultivars.

A third criterion for assessing marker‐based cultivar differences relied on the AMOVA, as proposed by Julier et al. (2018). For each of the 20 combinations of filtering levels of each genotyping method, Nei's (1972) distance matrix between pairs of cultivars was computed, and the AMOVA for each cultivar pair was performed using the function “stamppAmova” of the StAMPP R package version 1.6.3 (Pembleton et al., 2013) with the parameter “nperm = 10,000.” In this case, two cultivars were considered distinct if they differed at *p* < 0.05 in the AMOVA. The current adoption of a more liberal *p* value was justified by the fact that cultivar distinctness was based on a single statistical test, rather than several tests as in the case of several morphophysiological traits or PC axes (whose overall cultivar distinctness result implied a cumulation of *p* < 0.01 error rates). The correctness of the analysis was verified by obtaining R scripts and data used in the study that proposed this approach (Julier et al., 2018) and verifying the correct reproduction of the results.

One last criterion for assessing marker‐based cultivar distinctness reflected Q. Yu et al.’s (2022) criterion for the distinction of ryegrass varieties. They considered a cultivar as distinct from any other cultivar if its bulked DNA samples (replicates) were classified unequivocally and exclusively in the same group according to a probability threshold for group membership (shown at the node of the relevant group) set by bootstrap analysis within a hierarchical cluster analysis. Q. Yu et al. (2022) constructed the phylogenetic tree by the maximum likelihood method, using an algorithm that typically requires single‐individual sequencing and SNP dosage data, as in the case of MEGA (Hall, 2013), phangorn (Schliep et al., 2017), or RAxML‐NG (Kozlov et al., 2019). Therefore, they adapted their ryegrass bulk sequencing data to the standard framework by calling SNP dosages into 1, 0.5, and 0 classes. To express the actual allele frequency variation of the cultivars, we built the phylogenetic tree by the UPGMA (unweighted pair group method with arithmetic mean) method (Khan et al., 2008; Vaz et al., 2021) in a cluster analysis performed on the correlation‐based distance matrix constructed from allele frequency data. UPGMA and maximum likelihood methods displayed consistent phylogenetic trees in alfalfa when they were based on the same data (Yan et al., 2024). Our cluster analysis was repeated via bootstrapping with 1000 replications, estimating approximate probability values according to Shimodaira (2002). This procedure was implemented using the pvclust R package (Suzuki et al., 2019). Consensus phylogenetic trees for GBS and DArTag markers were constructed using the best marker configuration that emerged for the criterion based on ANOVAs on cultivar PC scores. We adopted a probability threshold ≥95% (analogous to a *p* < 0.05 error rate) for the exclusive classification of all cultivar replicates in the same group. Because of the absence of explicit pairwise comparisons, the results of this analysis were expressed only in terms of the number of cultivars that proved different from any other cultivar.

The consistency of cultivar diversity information provided by morphophysiological traits, GBS markers, and DArTag markers was verified by assessing the correlations between Euclidean distance matrices computed for each information layer by Mantel's test using the mantel() function of the R package “vegan” (Legendre & Legendre, 2012), averaging the results over 10,000 permutations. The consistency was also assessed graphically through a classical multidimensional scaling (MDS) analysis (alias principal coordinates analysis) based on cultivar Euclidean distances (Gower, 1966), displaying cultivar diversity values in a low‐dimensional space of two to four axes (2D or 4D). This analysis, which is recommended for displaying genotype diversity patterns in the presence of many original variables (as currently for molecular data) (Mohammadi & Prasanna, 2003), was performed using the cmdscale() function of the R package “stats.” Molecular marker‐based Euclidean distances were computed using the most discriminating marker configuration according to the criterion based on ANOVAs on cultivar PC scores (which included 17,937 polymorphic markers for GBS and 1770 polymorphic markers for DArTag).

## 3 RESULTS

### 3.1 Morphophysiological trait‐based distinctness

The morphophysiological evaluation of the 18 cultivars revealed wide phenotypic variation for plant height in spring and in autumn (PHS and PHA, respectively), which, for both traits, resulted in about 100 distinct pairs of cultivars out of 153 paired comparisons between the 18 cultivars (Table 1). These traits, however, were highly correlated to each other (*r* = 0.98; Table 2), as expected from the fact that both of them estimated the cultivar winter activity, that is, the ability to grow during the cool season (which is the opposite of autumn dormancy). Stem length and onset of flowering exhibited relatively high individual ability to distinguish cultivars (68 and 86 distinct pairs of cultivars, respectively; Table 1). However, even their information was largely redundant when added to that of any plant height trait associated with autumn dormancy, according to the correlations of taller plant with longer stem (*r* ≥ 0.82; Table 2) and earlier flowering (*r* ≤ −0.90; Table 2). All plant height or stem length traits were highly correlated to each other (*r* ≥ 0.82; Table 2) and provided, therefore, modest additional information for cultivar distinction when added to each other.

**TABLE 1:** Acronym, cultivar range values, and number and proportion of pairs of distinct cultivars (*p* < 0.01) in 153 paired comparisons among 18 alfalfa cultivars, for eight morphophysiological traits.

Trait	Acronym	Range	Number	Proportion (%)
Plant growth habit (scale 1–9)	PGH	1–3	10	6.5
Plant height in spring (cm)	PHS	16.8–49.2	104	68.0
Onset of flowering (no. of days from January 1)	OF	129–143	86	56.2
Proportion of plants with very dark flower	DFP	0.04–1.00	28	18.3
Proportion of plants with variegated flower	VFP	0.00–0.15	20	13.7
Length of tallest stem at full flowering (cm)	SL	89.5–130.0	68	44.4
Plant height 3 weeks after harvesting (cm)	PHH	31.4–54.2	34	22.2
Plant height in autumn (3 weeks after equinox; cm)	PHA	15.4–45.0	96	62.8

**TABLE 2:** Phenotypic correlation among eight morphophysiological traits observed on 18 alfalfa cultivars (see Table 1 for acronym of traits).

Trait	PHS	OF	DFP	VFP	SL	PHH	PHA
PGH	0.32	−0.07	−0.61**	0.10	0.39	0.27	0.29
PHS	–	−0.91***	−0.59**	0.12	0.82***	0.89***	0.98***
OF	–	–	0.33	−0.08	−0.62**	−0.74***	−0.90***
DFP	–	–	–	−0.35	−0.55*	−0.58*	−0.56*
VFP	–	–	–	–	0.19	0.04	0.09
SL	–	–	–	–	–	0.86***	0.84***
PHH	–	–	–	–	–	–	0.91***

*, **, and *** = different from zero at *p *< 0.05, *p *< 0.01, and *p *< 0.001, respectively.

**FIGURE 1:**
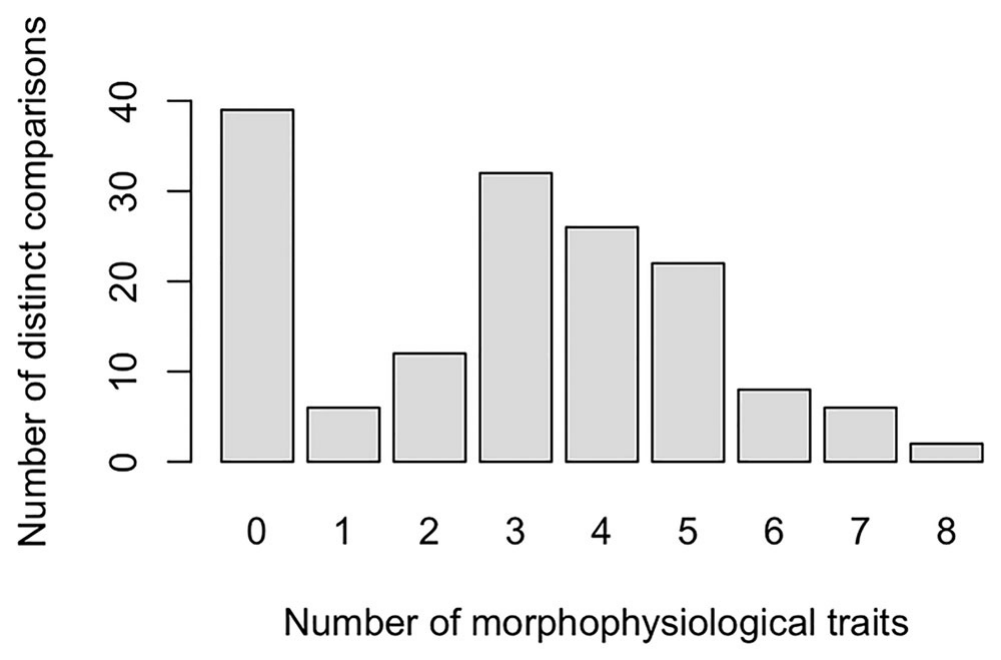
Number of pairwise comparisons among 18 alfalfa cultivars that showed distinctness (*p* < 0.01) for a number of morphophysiological traits ranging from zero (no distinctness) to eight.

Growth habit, frequency of variegated flower, and frequency of dark blue‐violet flower displayed low correlation with each other or with other traits (Table 2), along with modest discriminating ability (Table 1). The first two traits exhibited modest variation for ordinary ssp. *sativa* cultivars such as the current ones, which typically feature erect or semi‐erect growth habit and low frequency of variegated flower (a trait that derives from introgression from ssp. *falcata*). The wide range of variation observed for the frequency of dark blue‐violet flower (Table 1) was actually due to one cultivar possessing only dark flowers, while nearly all the other cultivars had low to fairly low frequency values. On the whole, 114 paired cultivar comparisons out of 153, that is, nearly 75%, displayed at least one difference (*p* < 0.01) for morphophysiological traits (Table 3). Eight pairs of cultivars were distinct according to seven or eight traits (Figure 1), all of them including the cultivar coded as K. Only three cultivars (F, K, and L), that is, nearly 17% of the 18 cultivars, exhibited distinctness from any other cultivar for at least one trait (Table 3).

**TABLE 3:** Number and proportion of (a) pairs of distinct cultivars in 153 paired comparisons among 18 alfalfa cultivars and (b) cultivars distinct from any other cultivar, according to different criteria.

	Distinct in paired comparisons		Distinct from any other	
Criterion (acronym)	Number	Proportion (%)	Number	Proportion (%)
Morphophysiological trait‐based distinctness (Morpho)^a^	114	74.5	3	16.7
GBS‐generated SNPs, LSD in ANOVA for PC axes (GBS‐PCA)^b^	142	92.8	9	50.0
DArTag SNPs, LSD in ANOVA for PC axes (DArTag‐PCA)^b^	142	92.8	11	61.1
GBS‐generated SNPs, PCA followed by discriminant analysis^c^	119	77.8	7	38.9
DArTag SNPs, PCA followed by discriminant analysis^c^	127	83.0	8	44.4
GBS‐generated SNPs, cluster analysis^d^	–	–	5	27.8
DArTag SNPs, cluster analysis^d^	–	–	9	50.0
Morpho + GBS‐PCA	147	96.1	10	55.6
Morpho + DArTag‐PCA	144	94.1	11	61.1

Abbreviations: ANOVA, analysis of variance; GBS, genotyping‐by‐sequencing; LSD, least significant difference; PC, principal component; PCA, principal component analysis; SNP, single nucleotide polymorphism.

^a^ Distinctness based on at least one least‐significance difference at *p* < 0.01 in analyses of variance for eight traits.

^b^ Polymorphic markers subjected to a principal components analysis followed by an analysis of variance performed on cultivar scores of individual PC axes; distinctness based on at least one least‐significance difference at *p* < 0.01.

^c^ Distinctness based on the correct classification of all bulked DNA samples in a discriminant analysis performed on cultivar principal component scores (using one sample per cultivar for validation).

^d^ Distinctness based on the unequivocal and exclusive classification of all bulked DNA samples of a cultivar in the same group with a bootstrap‐based probability ≥95% at the node of the relevant group.

### 3.2 Molecular marker‐based distinctness

The numbers of polymorphic markers available for GBS and DArTag markers are reported in Table S1 for the 20 configurations identified by different thresholds of maximum missing genotypes and minimum number of reads per marker. Such numbers ranged from 314 to over 27,000 for GBS markers and from 1210 to 1811 for DArTag markers, passing from the most stringent configuration (a maximum of 1% missing genotypes per marker; a minimum of 40 reads per marker) to the most liberal one (a maximum of 30% missing genotypes per marker; a minimum of 10 reads per marker).

For both genotyping methods, the marker configuration including a maximum of 30% missing genotypes per marker and a minimum of 20 reads per marker was the most discriminating on the basis of paired cultivar comparisons issued by an initial PCA followed by the assessment of cultivar differences in ANOVAs performed on cultivar PC scores. This configuration involved about 10‐fold greater marker number for GBS compared to DArTag markers (17,937 vs. 1770), as well as about 10‐fold greater average read depth per marker for DArTag relative to GBS markers (578.6 vs. 56.4). Both genotyping methods, when using their optimal configurations, displayed the same number of distinct pairs of cultivars, namely, 142 out of 153 (i.e., about 93%; Table 3). This result arose from ANOVAs on cultivar scores of the first 14 PC axes for GBS and the first 15 PC axes for DArTag markers. The PC 1 and PC 2 cultivar score values used for the ANOVAs are reported graphically in Figure S1 for both genotyping methods to show the occurrence of detectable variation among bulked DNA samples of the same cultivar even for the current case of 200 independent bulked genotypes. DArTag genotyping exhibited an advantage over GBS with respect to the number of cultivars that were distinct from any other cultivar, namely, 11 cultivars out of 18 (about 61%; cultivars B, C, E, F, G, I, J, K, L, N, and R) for DArTag, and nine out of 18 (50%) for GBS (Table 3).

For both genotyping methods, the criterion based on a discriminant analysis performed on cultivar PC scores was not as sensitive as that based on ANOVAs of cultivar PC scores for the detection of distinct cultivars (when considering their most discriminating marker configurations). The proportion of distinct cultivars in paired cultivar comparisons achieved by discriminant analysis approached 83% using DArTag markers and 78% using GBS markers (Table 3). Likewise, the number of completely distinct cultivars was lower according to this criterion, namely, eight according to DArTag markers and seven according to GBS markers (Table 3).

The criterion described in Julier et al. (2018) based on an AMOVA of marker data failed to detect any difference between paired cultivars at the *p* < 0.05 threshold for any of the 20 marker configurations of GBS or DArTag markers.

**FIGURE 2:**
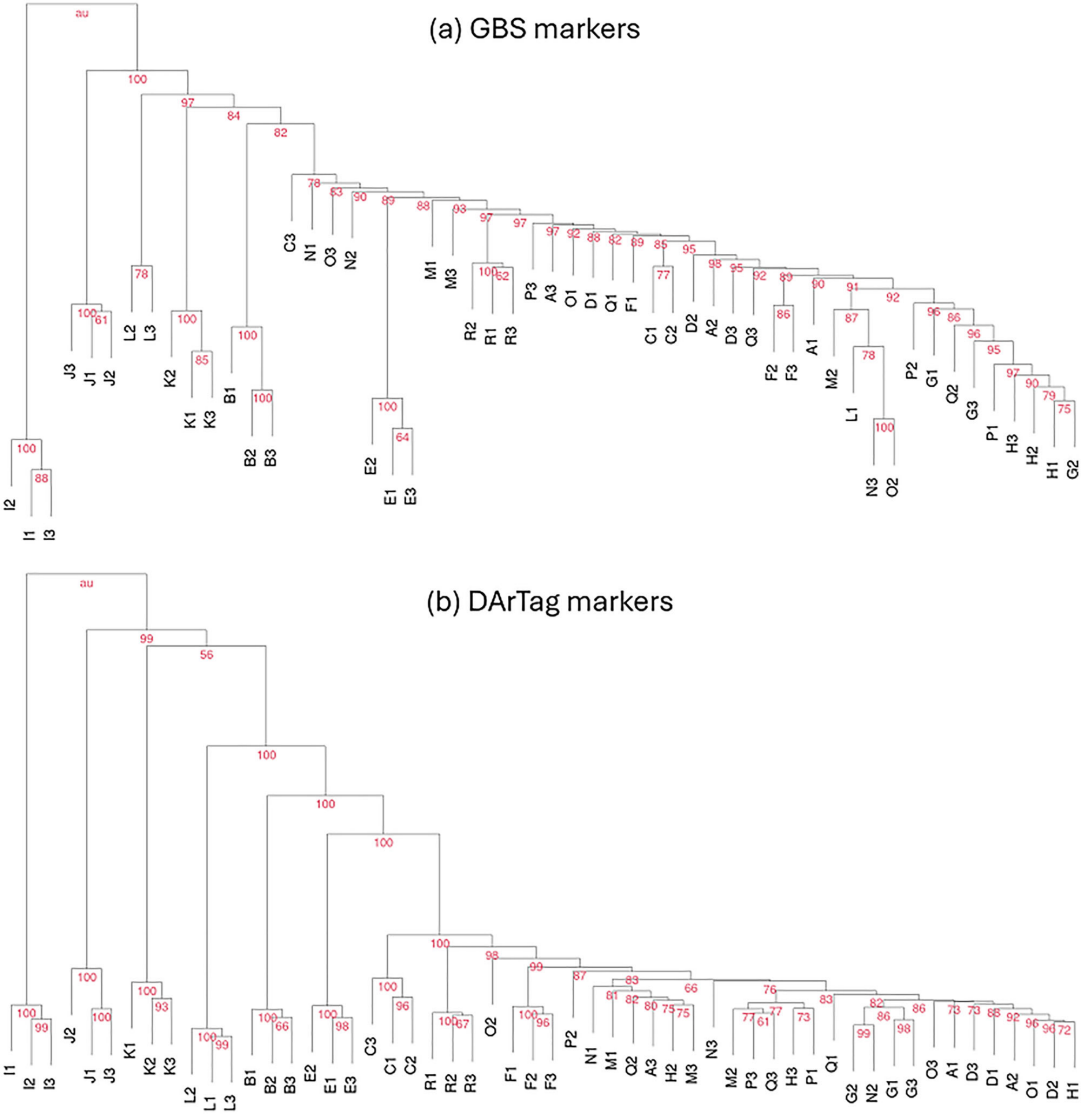
Hierarchical cluster analysis with percentage probability values from bootstrap analysis reported at the nodes, performed on Euclidean distances based on allele frequencies of 54 bulked DNA samples relative to 18 cultivars (coded by letters) and three samples per cultivar (indicated by numbers) for (a) 17,937 genotyping‐by‐sequencing (GBS)‐generated single nucleotide polymorphism (SNP) markers, and (b) 1770 DArTag SNP markers.

The criterion based on a hierarchical cluster analysis of the bulked DNA samples allowed the detection of nine completely distinct cultivars according to DArTag markers and five cultivars according to GBS markers (Table 3), on the basis of the unequivocal and exclusive classification of the bulked DNA samples of each cultivar in the same group with a bootstrap‐based probability ≥95% at the node of the relevant group (Figure 2). For example, the completely distinct cultivars according to DArTag markers were B, C, E, F, I, J, K, L, and R (Figure 2b). The heat map of the distance matrix constructed from allele frequency data that were used for the cluster analysis is reported in Figure S2 for both sets of markers. While this statistical criterion was less sensitive than that based on ANOVAs of cultivar PC scores for identifying completely distinct cultivars, its results confirmed the superiority of DArTag markers over GBS for the detection of completely distinct cultivars that emerged also for other statistical criteria (Table 3).

### 3.3 Combination of morphophysiological and molecular distinctness

**FIGURE 3:**
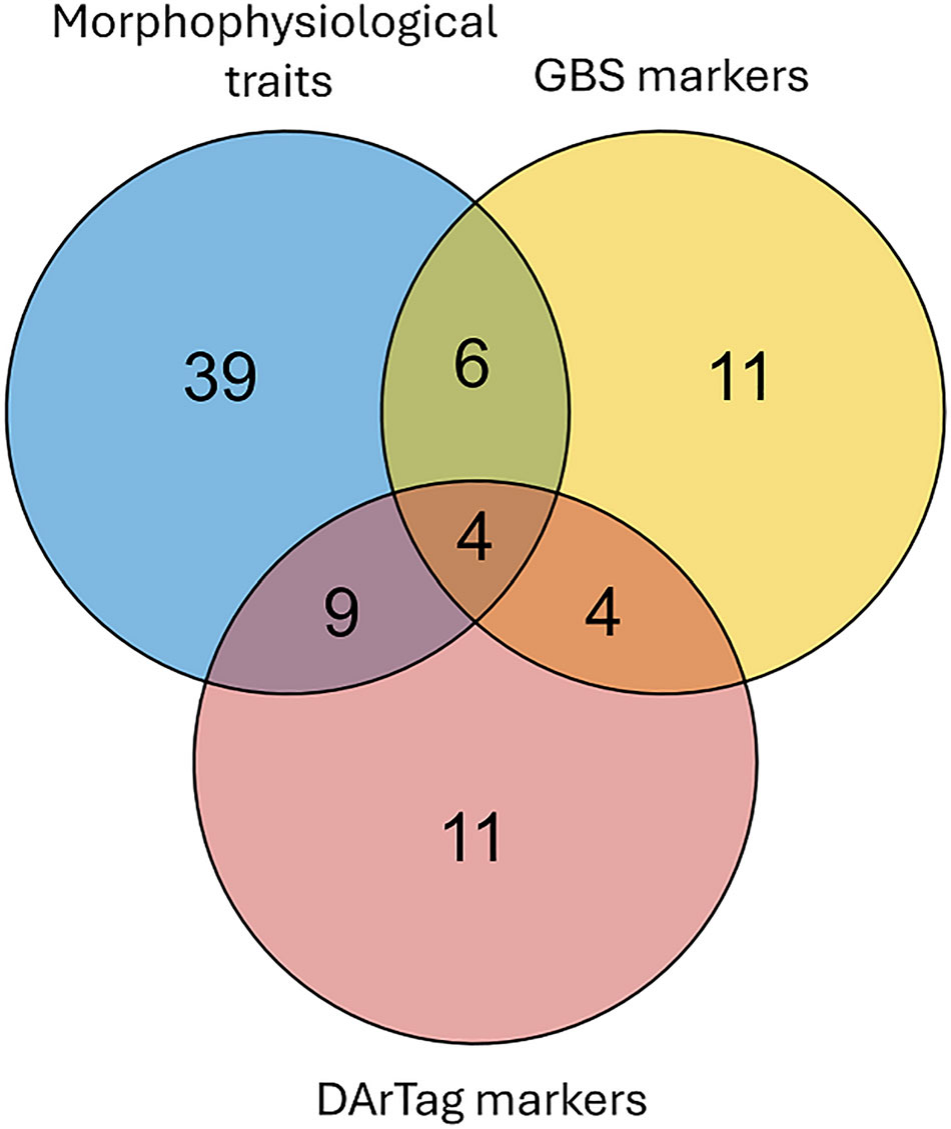
Number of non‐distinct pairs of cultivars (at the *p* < 0.01 threshold) in 153 paired comparisons among 18 alfalfa cultivars according to three distinctness criteria (morphophysiological traits; genotyping‐by‐sequencing [GBS] markers, or DArTag markers, subjected to analysis of variance of cultivar principal component scores) and their combinations.

This scenario was investigated only with respect to the most discriminating molecular marker‐based criterion (i.e., ANOVAs performed on PC score values of the cultivars). There were 107 paired cultivar comparisons that exhibited a statistically significant difference according to all criteria (morphophysiological, GBS‐based, DArTag‐based). For both genotyping methods, using the molecular distinctness as a support to the morphophysiological distinctness led to a sharp reduction in the number of non‐distinct cultivars in paired cultivar comparisons (from 39 solely on a morphophysiological basis to six when integrating GBS markers and nine when integrating DArTag markers; Figure 3). Likewise, the number of cultivars that were distinct from any other increased from only three, according to morphophysiological traits, to nine when adding GBS information and 11 when adding DArTag information (Table 3). It is worth noting that the number of distinct cultivars in paired comparisons, or the number of completely distinct cultivars, increased very little or not at all when combining morphophysiological and molecular distinctness in comparison with molecular distinctness alone (Figure 3). Indeed, all paired cultivar comparisons that were morphophysiologically distinct were also distinct according to one molecular criterion (nearly always according to both), while the reverse was not true. Combining GBS‐based and DArTag‐based distinctness reduced to four (i.e., 2.6%) the number of non‐distinct cultivars in the paired comparisons, which remained non‐distinct even when combining the three distinctness criteria (Figure 3).

### 3.4 Consistency of morphophysiological and molecular cultivar diversity

**FIGURE 4:**
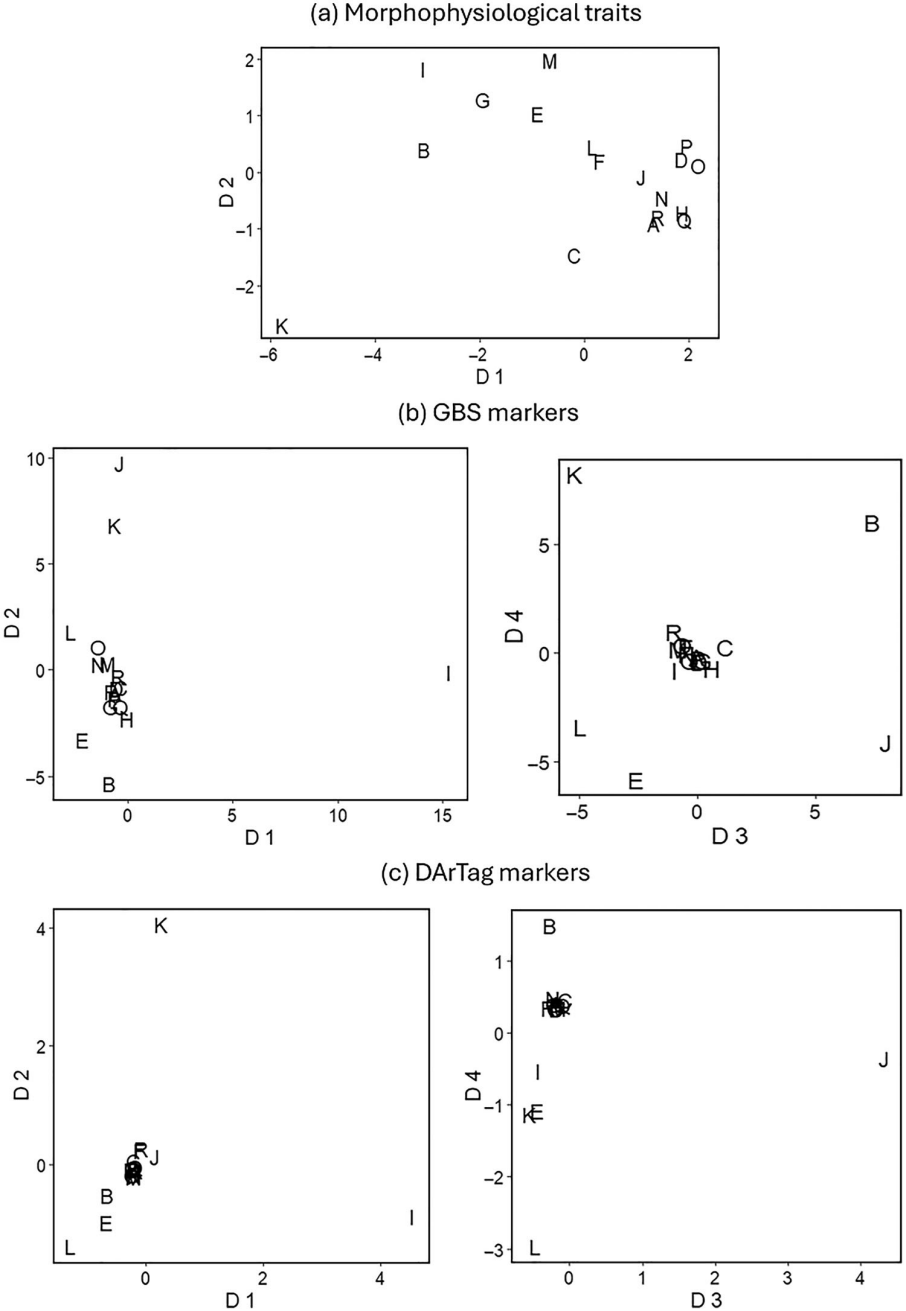
Ordination of 18 coded alfalfa cultivars along the first two to four dimensions (D) of a multidimensional scaling analysis performed on (a) eight morphophysiological traits, (b) 17,937 genotyping‐by‐sequencing (GBS)‐generated single nucleotide polymorphism (SNP) markers, and (c) 1770 DArTag SNP markers.

The ordination of the 18 coded cultivars along the first dimension of a MDS analysis is reported in Figure 4 for data layers relative to morphophysiological traits, GBS markers, and DArTag markers (using markers of the most discriminating configuration in the earlier analyses). A two‐dimensional representation could be considered adequate for morphophysiological data according to the stress statistics value of 0.22 (Dexter et al., 2018), while even the four reported dimensions were not fully adequate for marker data (given a stress statistics value of 0.61 for GBS markers and 0.49 for DArTag markers). Morphophysiological and molecular data unanimously highlighted the distinctness of the cultivar K. In addition, both genotyping methods revealed a genetic distinctness in the space of the first four dimensions for the cultivars J and I and, to a lesser extent, the cultivars B and L, all of which were much less (or hardly) evident according to morphophysiological traits. Mantel's test results revealed the high consistency between Euclidean distance measures of the cultivars outputted by GBS and DArTag markers (*r* = 0.96, *p* < 0.001) and the modest correlation (*p* < 0.05) of the distance measures based on morphophysiological traits with those issued by GBS (*r* = 0.46) or DArTag markers (*r* = 0.39).

## 4 DISCUSSION

Our study focused on the 18 most grown alfalfa varieties in Italy, considered as a highly relevant germplasm set because of their commercial importance, the leading position of Italy for the number of registered alfalfa varieties in Europe (with a share of 34%: EUPVP, 2024), and the convenience of focusing on varieties from a similar geographic origin (whose distinctness may be more challenging than that of varieties bred in geographically contrasting regions). It provides the first scientific evidence of the fact that the requirement of a registered variety to be “clearly distinguishable from any other variety” could hardly be satisfied even for already registered varieties of alfalfa when using the ordinary DUS criterion based on morphophysiological traits. Only about 17% of the cultivars exhibited complete distinctness, while about 25% of the paired comparisons between cultivars (39 out of 153) were non‐distinct, according to this criterion. The prior classification of all the current commercial varieties as completely distinct in earlier DUS trials was favored by the inclusion of just a small set of control varieties as testers. Indeed, a lower proportion of completely distinct cultivars would likely have emerged if we had been able to compare the several hundreds of alfalfa cultivars that are currently registered in EU member states (not to mention the around 1000 varieties listed in the OECD catalogue). Incidentally, our study may have overestimated the proportion of morphophysiologically distinct cultivars, owing to its assessment based on data of one crop cycle instead of two cycles (as prescribed by UPOV). Using data from two crop cycles may imply a larger error term in cultivar comparisons for quantitative traits, namely, the cultivar × crop cycle interaction, when the combined over‐years distinctness analysis shows significance for this interaction (UPOV, 2022). Quantitative traits are largely used for alfalfa cultivar distinctness, and our study highlighted their limitations due to high correlations among various traits relative to plant height, stem length, and onset of flowering. The observed modest ability of morphophysiological traits to distinguish commercial varieties may be even lower for candidate varieties, which ought to be distinct from all previously registered varieties.

Our results strongly reinforce the high potential importance of molecular distinctness that was advocated for outbred forage crops by Gilliland et al. (2020), indicating that the ordinary morphophysiological trait‐based distinctness (which was devised in an age featuring few registered varieties and no availability of molecular markers) is an inefficient and outdated criterion for alfalfa variety registration. This conclusion is supported by a 72% decrease (from 39 to 11; Figure 3) in the number of non‐distinct cultivars in pairwise comparisons and by a three‐fold to 3.7‐fold increase in the number of completely distinct cultivars (from 3 to 9 or 11; Table 3) when using GBS or DArTag marker‐based molecular distinctness instead of the morphophysiological distinctness. The combination of morphophysiological and molecular distinctness produced just a slight (or no) increase in the number of distinct varieties relative to molecular distinctness alone, since nearly all the morphophysiologically distinct cultivars were molecularly distinct according to both genotyping methods. Although highly valuable, even the molecular distinctness (alone or in combination with the morphophysiological distinctness) could ensure the complete distinctness from all other cultivars only for up to 61% of the cultivars (Table 3), further highlighting the challenge of cultivar distinctness in alfalfa. In substantial agreement with the current results, a prior study focusing on a set of 11 Italian alfalfa landraces reported a 75% decrease in the number of non‐distinct cultivars in pairwise comparisons passing from morphophysiological to GBS marker‐based distinctness and a modest additional decrease when combining the results of both criteria.

Molecular distinctness proved particularly effective as a support to ordinary DUS not only because of the wider cultivar diversity displayed by marker data relative to morphophysiological ones (as indicated by MDS analysis) but also because of the modest consistency between molecular and morphophysiological cultivar diversity highlighted by Mantel's test. This latter finding, while reinforcing the supporting role of molecular distinctness for ordinary DUS, suggests some caution when using molecular diversity as a proxy for morphophysiological diversity, as devised for the management of reference collections aimed to identify a small set of control cultivars with the greatest expected morphophysiological similarity with that of the candidate variety (UPOV, 2020). However, the fact that nearly all the morphophysiologically distinct cultivars were distinct according to both molecular criteria is also relevant for managing reference collections, suggesting that a marker‐based genetic distance could contribute, along with other information, to subset the number of control cultivars. The modest consistency between marker‐based and morphophysiological cultivar diversity agrees with results of several earlier studies relative to perennial forages such as alfalfa (Annicchiarico et al., 2016; Crochemore et al., 1998), white clover (Annicchiarico & Carelli, 2014; Kölliker et al., 2001), red clover (Dias et al., 2008; Greene et al., 2004; Pagnotta et al., 2011), perennial ryegrass (Roldán‐Ruiz et al., 2001), and tall fescue (Sun et al., 2015). A major exception to this general trend was the fairly high correlation between molecular and morphophysiological diversity reported for French alfalfa varieties by Julier et al. (2018).

DArTag markers, compared with GBS ones, allowed for better detection of completely distinct cultivars while possessing the following advantages: (a) about two‐third lower cost per genotyped cultivar (at least in the current study); (b) an open‐access technology; (c) a somewhat simpler SNP calling pipeline and lower amount of missing data (Table S1). Interestingly, the much lower number of polymorphic markers of the DArTag marker panel did not involve a lower amount of relevant genetic diversity—rather, a slightly opposite trend (confirmed also by the fact that there were 15 significant PC axes in the ANOVAs for DArTag markers vs. 14 for GBS markers). This result is remarkable, also in view of the fact that the panel set of 3000 SNP markers was selected from 40 cultivated alfalfa parents and founders from North American breeding programs (Zhao et al., 2023). Indeed, nearly 40% of its markers failed to display polymorphism for the current set of Italian cultivars. Most likely, the disadvantage of DArTag markers due to much lower marker number was counterbalanced by a very good marker dispersion across the genome and by better estimation of allele marker frequencies arising from about 10‐fold greater average read depth compared with GBS markers. Cultivar distinctness results for the 20 marker configurations indicated for both genotyping methods the usefulness of a minimum threshold of 20 reads per marker for allele frequency estimation. An accurate estimation of cultivar allele frequencies (which increases the ability to distinguish cultivars by minimizing the variation among cultivar replicates represented by bulked DNA samples) is also influenced by the sample of bulked plants. The current sample of 200 plants, already envisaged by Byrne et al. (2013), could not eliminate the variation among replicates of each cultivar (Figure S1) but contributed to decrease it. This hypothesis was verified indirectly for GBS markers by comparing the average root mean square error value of the ANOVAs performed on the first five PC axes in the current study, which was equal to 0.67, with that of an earlier alfalfa study (Annicchiarico et al., 2016) based on 100 plants per bulked DNA sample, which amounted to 4.83.

The adopted statistical criterion affected the results of the molecular distinctness assessment. The criterion based on ANOVAs performed on cultivar PC score values, which displayed a slight advantage over that based on the discriminant analysis in a prior study (Annicchiarico et al., 2016), currently showed the greatest sensitivity for detection of distinct cultivars. Its statistical threshold for variety difference (which may also affect the results) was currently set to *p* < 0.01 for consistency with the threshold recommended by UPOV (2022) for ANOVAs performed on morphophysiological traits. The statistical criterion based on cluster analysis with bootstrap values, which could offer a more explicit graphical representation of the cultivars that were completely distinct, ranked second for sensitivity to detect such cultivars based on DArTag markers (Table 3). The criterion based on discriminant analysis, which was nearly as valuable as cluster analysis in this respect, could contemplate a statistically significant distinctness only in the case of no misclassification for the six validation samples of each paired cultivar comparison (one misclassification being equivalent to a 16.7% error rate). The complete lack of cultivar discrimination at *p* < 0.05 shown by the AMOVAs was unexpected and was influenced by the low number of bulked DNA samples per cultivar acting as replicates in the analysis. We verified this hypothesis by reanalyzing the data by Julier et al. (2018), who could distinguish 20 alfalfa commercial varieties by AMOVA‐based paired comparisons with a *P* level in the range of 0.025–0.033 in a study including four bulked DNA samples per cultivar. We found no difference at *p* < 0.05 between paired cultivars in their dataset when the AMOVAs included only three bulked samples per cultivar (selected randomly) instead of four. In general, the results of our comparison of statistical criteria were affected by the experiment layout (particularly in terms of the number of bulked DNA samples) and the fixed *P* levels, and are susceptible to change for other scenarios.

## 5 CONCLUSIONS

Our results strongly encourage the introduction of alfalfa molecular distinctness as a support to ordinary DUS to increase the distinctness of the registered cultivars and to decrease the number of candidate varieties that are rejected from registration while possessing sufficient VCU (thereby benefitting from the agronomic progress and the resource investment of these varieties). The implementation of molecular distinctness could conveniently rely on DArTag markers and, for Europe, be preceded by the molecular characterization of the entire European reference collection. Based on our findings, anyway, this collection would include a subset of registered cultivars that could not be distinguished even on a molecular basis. The adoption of molecular distinctness involves additional, remarkable advantages relative to morphophysiological distinctness in terms of test duration (some months vs. 3 years), cost, and reliability of the assessment (with respect to genotype × environment interactions affecting quantitative traits). For example, the molecular assessment could be verified at the very beginning of the variety registration process, providing feedback sufficiently timely to prevent the onset of the VCU assessment (or at least its prolongation beyond the first year) in the absence of acceptable variety distinctness. Anyway, plant breeders could conveniently verify the molecular distinctness of a candidate variety before its proposition for registration, assuming that molecular data for registered cultivars are made publicly available. Other important potential advantages of the molecular distinctness concern its timely application in two important contexts, namely, forensic analyses for lawsuits (where the variety protection is practically enforced) and the control of certified seed. Officers charged with field inspections for certified seed production in Italy repeatedly reported the impossibility of verifying the identity of alfalfa varieties under multiplication on the basis of morphophysiological traits (G. Spataro, personal communication, 2024), while the morphophysiological trait‐based control of random samples of certified seed lots suffers from the same time and cost limitations as the DUS assessment. The introduction of a molecular marker‐based assessment could increase the sensitivity, rapidity, and cost‐efficiency of the control of certified seed. As anticipated, the risk of variety plagiarism is hardly relevant for molecular distinctness based on the overall genetic diversity for a very large marker number. While initially established as a support to ordinary DUS according to Gilliland et al.’s (2020) proposal, molecular distinctness may ultimately replace the morphophysiological distinctness and encompass the entire DUS assessment if emphasis was placed on genetic differences rather than morphophysiological ones and a reliable use of markers also for uniformity and stability assessments could be devised.

Our results concern alfalfa, but similar limitations of ordinary DUS and opportunities for molecular distinctness may emerge for other major perennial forages bred as synthetic varieties. The peculiar challenges of DUS for these species encourage other similar studies and urge a joint reflection of breeders and variety registration offices aimed to set up original and effective solutions that can live up to the needs of modern agriculture. As a first step in the direction of expanding the criteria for granting variety distinctness in forage crops, the Community Plant Variety Office has recently allowed the use of seed protein polymorphisms for DUS of perennial ryegrass varieties in case of lacking morphophysiological distinctness (CPVO, 2019).

## AUTHOR CONTRIBUTIONS

**Paolo Annicchiarico**: Conceptualization; funding acquisition; methodology; supervision; visualization; writing—original draft. **Nicolò Franguelli**: Formal analysis; investigation; writing—review and editing. **Barbara Ferrari**: Investigation; writing—review and editing. **Giacomo Campanella**: Investigation. **Stefano Gualanduzzi**: Investigation. **Margherita Crosta**: Formal analysis. **Chiara Delogu**: Funding acquisition; project administration; writing—review and editing. **Giorgia Spataro**: Data curation. **Nelson Nazzicari**: Data curation; formal analysis; investigation; methodology; software; visualization; writing—review and editing.

## CONFLICT OF INTEREST STATEMENT

The authors declare no conflicts of interest.

## Supporting information




**Supplemental Table S1**. Number of polymorphic GBS‐generated and DArTag SNP markers as a function of different thresholds of maximum missing genotypes per marker (mpm) and minimum reads per marker (rm).

**Supplemental Fig. S1**. Ordination of bulked DNA samples of 18 coded alfalfa cultivars (three samples per cultivar) along the first two axes (PC 1 and PC 2) of a principal components analysis performed on (a) 17,937 GBS‐generated SNP markers and (b) 1,770 DArTag SNP markers.

**Supplemental Fig. S2**. Heat map of the correlation‐based distance matrix constructed from allele frequency data for 54 bulked DNA samples relative to 18 cultivars (coded by letters) and three samples per cultivar (indicated by numbers) for (a) 17,937 GBS‐generated SNP markers, and (b) 1,770 DArTag SNP markers.

## Data Availability

Morphophysiological trait data, and GBS and DArTag SNP marker data, relative to their most discriminating configurations, are publicly available in the Figshare repository at https://doi.org/10.6084/m9.figshare.27960579.
