# Supplementary material for: Molecular markers enhance substantially the distinctness of alfalfa varieties for registration and protection
Source: Plant Genome. 2025 Feb 5;18(1):e20556. doi: 10.1002/tpg2.20556 (PMC11795343; doi:10.1002/tpg2.20556)
Supplement: Supplementary file 1 [file TPG2-18-e20556-s001.docx]

Paolo Annicchiarico, Nicolò Franguelli, Barbara Ferrari, Giacomo Campanella, Stefano Gualanduzzi, Margherita Crosta, Chiara Delogu, Giorgia Spataro, and Nelson Nazzicari - Molecular Markers Enhance Substantially the Distinctness of Alfalfa Varieties for Registration and Protection

**Supplemental Table S1.** **Number of polymorphic GBS-generated and DArTag SNP markers as a function of different thresholds of maximum missing genotypes per marker (mpm) and minimum reads per marker (rm).**

|  | **rm, GBS-generated SNPs** | | | |  | **rm, DArTag SNPs** | | | |
| --- | --- | --- | --- | --- | --- | --- | --- | --- | --- |
| **mpm** | **10** | **20** | **30** | **40** |  | **10** | **20** | **30** | **40** |
| 0.01 | 8125 | 3098 | 1094 | 314 |  | 1344 | 1306 | 1264 | 1210 |
| 0.05 | 13019 | 6637 | 3076 | 1290 |  | 1533 | 1504 | 1471 | 1441 |
| 0.10 | 17169 | 9989 | 5895 | 2994 |  | 1624 | 1601 | 1569 | 1535 |
| 0.20 | 22970 | 14515 | 10245 | 6998 |  | 1729 | 1698 | 1664 | 1640 |
| 0.30 | 27078 | 17937 | 13341 | 10175 |  | 1799 | 1770 | 1744 | 1713 |
