# Supplementary figures and images for: Molecular markers enhance substantially the distinctness of alfalfa varieties for registration and protection
Source: Plant Genome. 2025 Feb 5;18(1):e20556. doi: 10.1002/tpg2.20556 (PMC11795343; doi:10.1002/tpg2.20556)

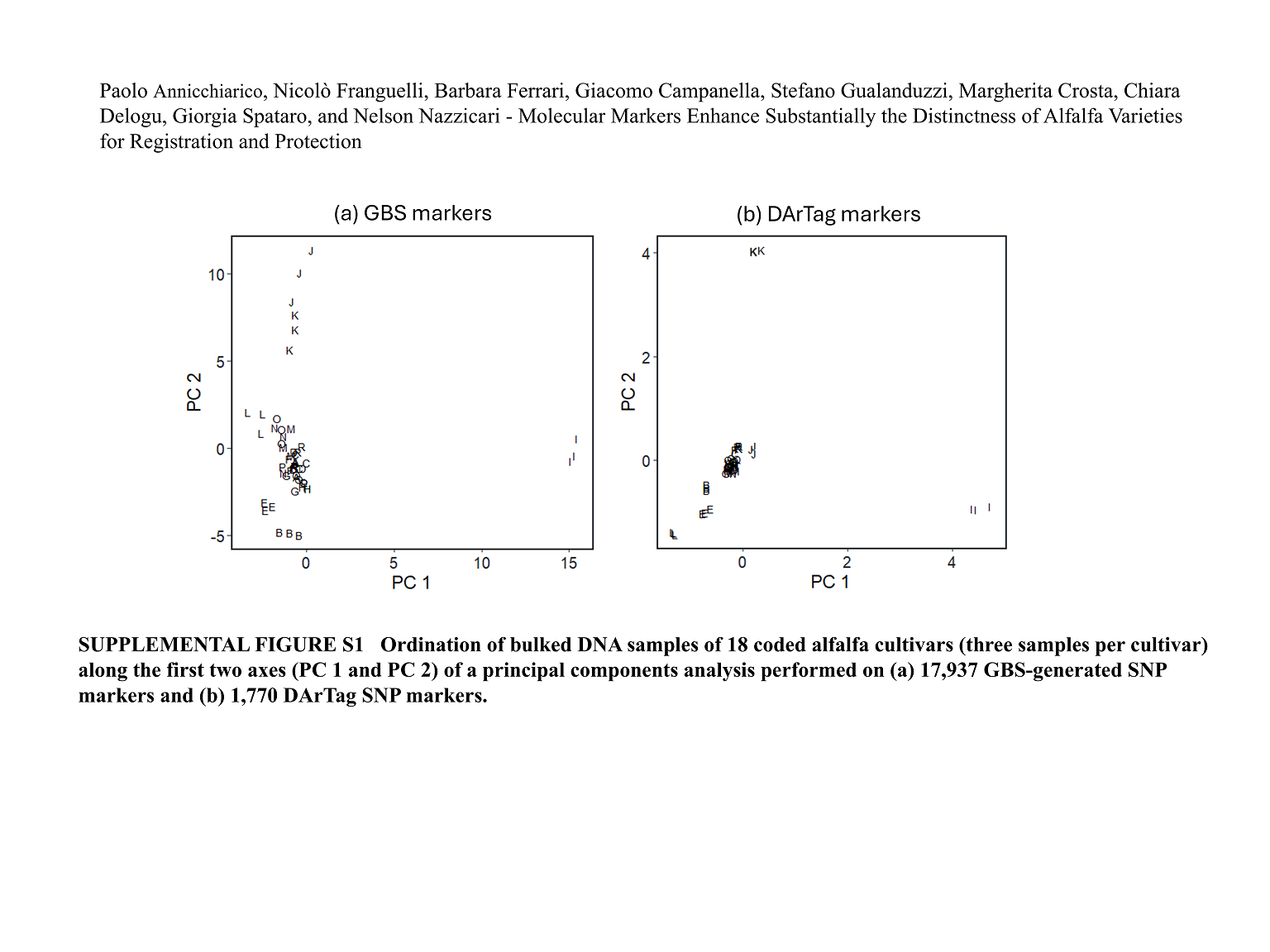

Supplement: Supplementary file 2 — Supplemental Fig. S1. Ordination of bulked DNA samples of 18 coded alfalfa cultivars (three samples per cultivar) along the first two axes (PC 1 and PC 2) of a principal components analysis performed on (a) 17,937 GBS‐generated SNP markers and (b) 1,770 DArTag SNP markers. [file TPG2-18-e20556-s003.tif]

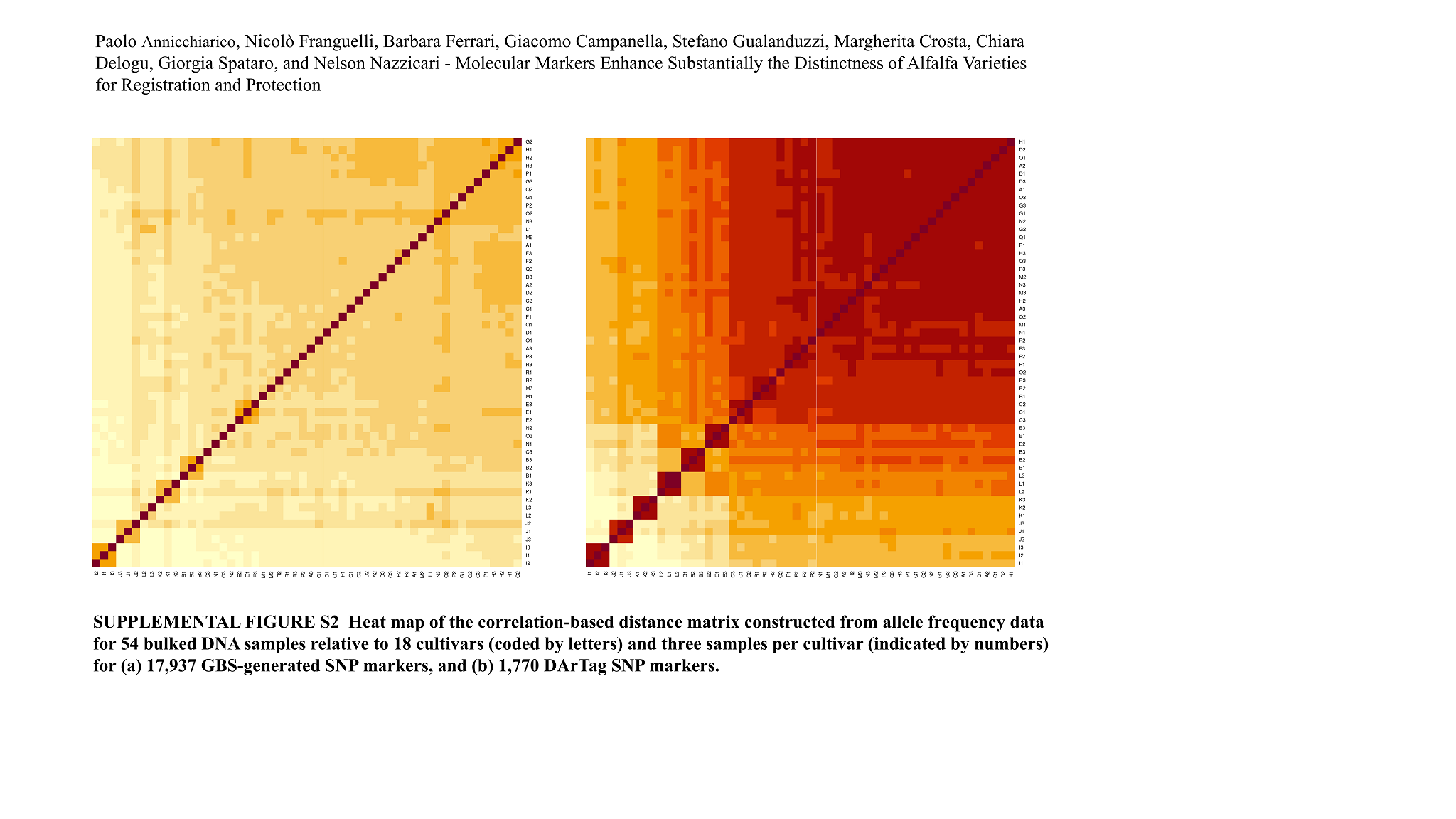

Supplement: Supplementary file 3 — Supplemental Fig. S2. Heat map of the correlation‐based distance matrix constructed from allele frequency data for 54 bulked DNA samples relative to 18 cultivars (coded by letters) and three samples per cultivar (indicated by numbers) for (a) 17,937 GBS‐generated SNP markers, and (b) 1,770 DArTag SNP markers. [file TPG2-18-e20556-s002.tif]
